# Therapeutic Potential and Limitation of Serotonin Type 7 Receptor Modulation

**DOI:** 10.3390/ijms24032070

**Published:** 2023-01-20

**Authors:** Kouji Fukuyama, Eishi Motomura, Motohiro Okada

**Affiliations:** Department of Neuropsychiatry, Division of Neuroscience, Graduate School of Medicine, Mie University, Tsu 514-8507, Japan

**Keywords:** antipsychotics, antidepressants, mood stabilising, cognition, lurasidone, schizophrenia, serotonin type 7 receptor, vortioxetine

## Abstract

Although a number of mood-stabilising atypical antipsychotics and antidepressants modulate serotonin type 7 receptor (5-HT7), the detailed contributions of 5-HT7 function to clinical efficacy and pathophysiology have not been fully understood. The mood-stabilising antipsychotic agent, lurasidone, and the serotonin partial agonist reuptake inhibitor, vortioxetine, exhibit higher binding affinity to 5-HT7 than other conventional antipsychotics and antidepressants. To date, the initially expected rapid onset of antidepressant effects—in comparison with conventional antidepressants or mood-stabilising antipsychotics—due to 5-HT7 inhibition has not been observed with lurasidone and vortioxetine; however, several clinical studies suggest that 5-HT7 inhibition likely contributes to quality of life of patients with schizophrenia and mood disorders via the improvement of cognition. Furthermore, recent preclinical studies reported that 5-HT7 inhibition might mitigate antipsychotic-induced weight gain and metabolic complication by blocking other monoamine receptors. Further preclinical studies for the development of 5-HT7 modulation against neurodevelopmental disorders and neurodegenerative diseases have been ongoing. To date, various findings from various preclinical studies indicate the possibility that 5-HT7 modifications can provide two independent strategies. The first is that 5-HT7 inhibition ameliorates the dysfunction of inter-neuronal transmission in mature networks. The other is that activation of 5-HT7 can improve transmission dysfunction due to microstructure abnormality in the neurotransmission network—which could be unaffected by conventional therapeutic agents—via modulating intracellular signalling during the neurodevelopmental stage or via loss of neural networks with aging. This review attempts to describe the current and novel clinical applications of 5-HT7 modulation based on preclinical findings.

## 1. Introduction

Serotonin (5-HT) receptor type 7 (5-HT7) is one of the most recently (1993) identified members of the 5-HT receptor family [1,2,3,4,5]. It has been demonstrated that 5-HT7 is highly expressed in functionally relevant regions of the brain [6,7]. Indeed, in the central nervous system, 5-HT7 is most predominantly expressed in the thalamus, hypothalamus, hippocampus, prefrontal cortex, basal ganglia, amygdala and dorsal raphe nucleus [8,9,10,11,12,13,14]. The predominant expression of 5-HT7 in the limbic regions provides a candidate hypothesis that 5-HT7 contributes to the regulation of memory processing, cognition and emotional perception [9,10,11,12,15,16]. The expression of 5-HT7 has been also observed in the kidney, liver, pancreas, spleen, stomach and smooth muscle cells of the arteries and gastrointestinal tract [17]. Based on these findings, 5-HT7 modulation is also considered to be a possible therapeutic target for the treatment of peripheral organs [18,19,20,21].

A number of preclinical studies have reported that 5-HT7 plays important roles in the regulation of mood, memory processing, cognition and emotional perception by following various experiments using selective 5-HT7 modulators and 5-HT7 knockout mice models [15,22,23,24,25,26]. Although modulating 5-HT7 is one of the targets for the treatment of schizophrenia and mood and anxiety disorders in current psychopharmacology, unfortunately, the clinical application of selective 5-HT7 receptor modulators has not yet been achieved [16]. However, several conventional mood-stabilising atypical antipsychotics, such as aripiprazole, brexpiprazole, clozapine, lurasidone, olanzapine, quetiapine, risperidone and zotepine are known to be inhibitors of 5-HT7 [12,27,28,29,30,31,32,33,34,35,36,37,38,39,40] (Table 1). Lurasidone is an antipsychotic agent with the highest binding affinity to 5-HT7 among mood-stabilising atypical antipsychotics [16,27] (Table 1). Furthermore, a novel antidepressant, vortioxetine, which is categorized as a 5-HT partial agonist reuptake inhibitor (SPARI), exhibits distinct pharmacodynamic profiles compared to other monoamine transporter-inhibiting antidepressants, since vortioxetine acutely and chronically suppresses the function of 5-HT7 [38,39,41,42]. Therefore, in order to clarify crucial clinical targets of 5-HT7 modulation for the treatment of several neuropsychiatric disorders, in the first half of this review we discuss the psychopharmacological perspectives of 5-HT7 modulation, based on the preclinical and clinical findings regarding 5-HT7 modulators and the clinical evaluation of lurasidone and vortioxetine to date.

Recent pharmacodynamic studies demonstrated that 5-HT7 modulation possibly contributes to a part of the characteristics of the clinical effects of both lurasidone and vortioxetine via inter-neuronal transmission [12,16,39,40,41,42,43]. Furthermore, recent preclinical findings suggest several possibilities in which 5-HT7 modulation can contribute to novel therapeutic targets for the treatment of several neuropsychiatric disorders, such as neurodevelopmental disorder, neurodegenerative diseases and metabolic complication associated with antipsychotics, through modulating intracellular signalling [5,42,44,45]. These candidate therapeutic potentials of 5-HT7 modulation are based on the findings that activated 5-HT7 enhances intracellular signalling, leading to synaptic remodelling and dendritic spine elongation [46]. In the second half of this review, we outline the recent pathophysiological hypotheses and their limitations (challenges to clarify in the future) regarding neurodevelopmental disorder and neurodegenerative diseases, based on the effects of 5-HT7 on intracellular signalling. In the final section, we introduce the possibility that inhibition of 5-HT7 is involved in the suppression of weight gain or metabolic complication induced by atypical antipsychotics via modulation of intracellular signalling [12,16,39,40,41,42,43,47,48].

**Table 1:** Receptor-binding profiles of antipsychotics and antidepressants.

Receptor	LUR	APZ	Brex	CLZ	OLZ	PMZ	QTP	RIS	ZTP	VTX
5-HT1A	6.8	5.6	0.12	124	>1000	650	432	423	471	15.0
5-HT2A	2.0	8.7	0.47	5.4	2.3	48.4	100	0.2	2.7	
5-HT3	>1000	630		241	57	>1000	>1000	>1000	472	3.7
5-HT7	0.5	10.3	3.7	18.0	365	0.5	307	6.6	12.0	19.0
H1	>1000	27.6	19	1.13	1.2	692	11	20.1	3.21	
D1	262	>1000	160	266	100	>1000	712	244	71.0	
D2	1.7	3.3	0.3	157	52.3	0.3	245	3.6	25.0	
Reference	[27]	[28,29]	[30]	[31,32]	[33,49]	[34]	[35]	[29,36]	[37]	[38]

Notes: lurasidone (LUR), aripiprazole (APZ), brexpiprazole (Brex), clozapine (CLZ), olanzapine (OLZ), pimozide (PMZ), quetiapine (QTP), risperidone (RIS), zotepine (ZTP) and antidepressant vortioxetine (VTX) against serotonin (5-HT) type 1A (5-HT1A), type 2A (5-HT2A), type 3 (5-HT3), and type 7 (5-HT7) receptor, histamine H1 (H1) receptor, dopamine receptors type 1 (D1) and 2 (D2). Data are equilibrium-constant (Ki) values (nM).

## 2. Preclinical Findings about Therapeutic Potential of 5-HT7 Modulation

### 2.1. Depression

The earliest identified physiological function of 5-HT7 was regulation of circadian rhythms [2]. In this context, 5-HT7 was found to be expressed in the suprachiasmatic nucleus [50], which is a major regulatory region of circadian rhythms [51]. The influence of 5-HT7 on sleep regulation is complicated because the 5-HT7 inverse agonist SB269970 [40,42,43,52] increased latency in the onset but decreased the total amount of time spent in rapid eye movement sleep [53]. A number of antidepressants also increased latency in the onset but decreased the total amount of time spent in rapid eye movement sleep, similar to SB269970 [54]. Based on these similar effects of SB269970 and antidepressants on sleep, exploration for antidepressant-like effects of 5-HT7 inhibitor was conducted.

The 5-HT7 knockout mice displayed a reduction of immobility times in both a forced swim test and a tail suspension test, common pharmacological behaviour tests for the screening of antidepressants (decreasing immobility time is considered to correlate to antidepressant action in humans) [22,55]. Based on this finding, 5-HT7 inhibitors/antagonists were actively explored and developed as antidepressants in the early years of this century. SB269970, an inverse agonist of 5-HT7, has been shown to reduce immobility time in forced swim and tail suspension tests; as a result, it became a candidate for use as an antidepressant [22,23,56]. Additionally, the administration of subeffective concentration of SB269970 enhanced the anti-immobility action of subeffective doses of of desipramine, citalopram and imipramine in the forced swim and tail suspension tests [56,57,58]. Most notably, SB269970 generated significantly rapid onset of antidepressant-like effects in olfactory bulbectomised models, when compared to fluoxetine [59]. As a result, 5-HT7 inhibitors were anticipated to join the rapid onset antidepressant class, since it is recognised that one of the major problems of monoamine transporter-inhibiting antidepressants, a currently/commonly prescribed antidepressant class, is that they require 2-4 weeks for the onset of antidepressive effects. The target regions for antidepressant effects of 5-HT7 inhibitors remain controversial. Injection of SB269970 into the hippocampus generated antidepressant-like activity in the forced swim test [60]. Injection of SB269970 into the lateral habenular nucleus lesion model using 6-hydroxydoapmine also exhibited antidepressant-like activity, whereas injection into the medial prefrontal cortex lesion of a model, conversely, displayed the enhancement of depressive-like activity [61,62]. AS19, a 5-HT7 agonist, demonstrated the opposite region-dependent effect against SB269970 [61,62] (Table 2).

### 2.2. Anxiety

The potential function of 5-HT7 on anxiety demonstrated the inconsistent results between pharmacological and molecular biological studies, similar to the case of depression. Expression of 5-HT7 mRNA increased by acute restraint stress but not by chronic variable stress in the rat hippocampus, indicating that 5-HT7 contributes to the regulation of response to stress [63]; however, the anxiety-like behaviour in the light/dark transfer or elevated plus maze tests did not affect both 5-HT7 knockdown or knockout models [55,64,65]. On the other hand, the anxiolytic-like activity of SB269970 (both systemic and intra-hippocampal local administrations) was demonstrated by the Vogel drinking test, the elevated plus maze test and the four plates test [23,60]. Both SB269970 and 5-HT7 knockout mice demonstrated anxiolytic-like activity in the marble-burying test, which is a model of anxiety and obsessive–compulsive disorder [66]. These inconsistent results among molecular biological and pharmacological experiments suggest that the level of 5-HT7 inactivation required for anxiolytic effects is probably dependent on the model utilized. In other words, appropriate 5-HT7 inhibition may be beneficial for anxiolytic effects (Table 2).

### 2.3. Schizophrenia

A number of pharmacological studies reported the therapeutic potential in components of positive and negative symptoms and cognitive dysfunction of schizophrenia using chemical-induced schizophrenia models. Compared with wild-type mice, 5-HT7 knockout mice were less susceptible to prepulse inhibition deficits of phencyclidine [67]. SB-269970 suppressed hyperactivity induced by ketamine, phencyclidine and amphetamine [68,69], whereas the effects of 5-HT7 on prepulse inhibition deficits were inconsistent. SB-269970 improved amphetamine-induced prepulse inhibition deficits [69], but did not affect those induced by an NMDA/glutamate inhibitor, phencyclidine or ketamine [67,70]; however, SB-258741, a 5-HT7 antagonist, did not affect the amphetamine-induced prepulse inhibition deficits, but suppressed phencyclidine-induced prepulse inhibition deficits [71]. Ketamine-induced negative symptoms associated with social withdrawal were attenuated by SB269970, but not affected by SB-258741 [70,71]. The effects of SB269970 on positive symptoms are possibly involved in dopaminergic transmission but not in glutamatergic transmission, whereas, conversely, the effects of SB-258741 on positive symptoms are possibly involved in glutamatergic transmission but not in dopaminergic transmission. Furthermore, social withdrawal induced by NMDA/glutamate receptor inhibition is prevented by SB269970 but not by SB258741. These discrepancies between SB269970 and SB258741 could not be explained by their receptor-binding profiles alone, since these compounds displayed binding affinity to 5-HT7, D2 and D3 receptors [71]. These findings suggest that the effects between SB269970 and SB258741 are probably dependent upon the materials (pharmacological and molecular biological models) and compounds employed (Table 2).

On the other hand, the effects of 5-HT7 inhibition on neurocognitive dysfunction (procognitive effects) demonstrated its promise. SB269970 attenuates amnesia in short-term memory induced by ketamine and MK801 [72,73], and this effect was suppressed by AS19 [74]. The new valuable tool for exploring the neurobiological bases of cognitive dysfunction in schizophrenia, five-choice serial reaction time task (including attention, response inhibition, cognitive flexibility and processing speed), demonstrated that SB269970 improved the impairment of working memory and impulsivity, without affecting premature responding induced by MK801 [75] (Table 2).

**Table 2:** Preclinical data on the role of 5-HT7 in depression, anxiety and schizophrenia.

Model	Agent Manipulation	Effects	Ref.
**Depression**			
Forced swim	Knockout	Decreased immobility	[22,55]
	SB269970	Decreased immobility (systemic administration)	[22,23]
		Decreased immobility (hippocampus)	[60]
6-OH-dopamine lesions (medial forebrain bundle)	AS-19 SB269970	Decreased immobility (into PrL) Increased immobility (into PrL) (Prl: the prelimbic subregion of the ventral medial prefrontal cortex)	[61]
Tail suspension	Knockout	Decreased immobility	[22]
	SB269970	Decreased immobility	[22,23,56]
Olfactory bulbectomy	SB269970	Decreased hyperactivity	[59]
**Anxiety**			
Elevated plus maze	Knockout Knockdown	No difference between models and wild-type	[55,64,65]
	SB269970	Increased time in open arms and entries into open arms	[23,60]
Light/dark transfer	Knockout Knockdown	No difference between models and wild-type	[55,64,65]
Vogel drinking Four plates test	SB269970	Increased the number of the accepted shocks Increased the number of punished crossings	[23,60]
Marble-burying	Knockout SB269970	Reduced stereotypic behaviour	[66]
**Schizophrenia**			
Prepulse inhibition	Knockout	Less susceptible to prepulse inhibition deficits by phencyclidine	[67]
	SB269970	Improved amphetamine-induced prepulse inhibition deficits	[69]
		No effects on prepulse inhibition deficits induced by phencyclidine/ketamine	[67,70]
	SB258741	No effect on amphetamine-induced prepulse inhibition deficits	[71]
		Suppressed phencyclidine-induced prepulse-inhibition deficits	[71]
	SB269970	Suppressed hyperactivity induced by ketamine, phencyclidine and amphetamine	[68,69]
Social withdrawal	SB269970	Improved	[70,71]
	SB258741	No effect	[70,71]
Amnesia induced by ketamine/MK801	SB269970	Improved	[72,73]
5-choice serial reaction time task	SB269970	Improved working memory impairment and impulsivity	[75]

## 3. Clinical Evaluation of 5-HT7 Modulators

### 3.1. Vortioxetine

Vortioxetine is an antidepressant belonging to the family of monoamine transporter-inhibiting antidepressants; its antidepressant effect is thought to arise from not only its monoamine transporter inhibition but also 5-HT7 inhibition. The binding affinity of vortioxetine to 5-HT7 is relatively low compared to serotonin transporter, 5-HT1A and 5-HT3 [38], but the relevant therapeutic concentration of vortioxetine functionally suppresses 5-HT7 [39,41] (Table 1).

The delay of the onset of the antidepressive effects of conventional monoaminergic antidepressant medication has been established as one of the major drawbacks of current treatments for depressive disorder, since all conventional monoamine transporter-inhibiting antidepressants require more than several weeks for the onset of beneficial antidepressive effects [76,77]. Based on the fast-acting antidepressant-like effects of 5-HT7 inhibitor SB269970 [59], a number of psychiatrists initially expected the rapid-onset antidepressant class to be prescribed in clinical settings in place of conventional monoamine transporter-inhibiting antidepressants; however, unfortunately, the onset duration of the antidepressant effect of lurasidone and vortioxetine is equivalent to that of conventional antidepressants [78,79,80,81,82]. The T1/2 value of vortioxetine is long (66 hrs), with steady-state plasma concentration levels reached after approximately 2 weeks, which may delay the onset of beneficial pharmacological effects of vortioxetine [83].

It has been recognized that comorbidity with anxiety (disorder) is the most significant factor affecting poor prognostic outcomes, such as increasing risk of recurrence, chronic course and suicidal behaviour [84,85]. Several meta-analysis studies have reported inconsistent results for the efficacy of vortioxetine on anxiety symptoms. A meta-analysis supported the efficacy of vortioxetine for generalised anxiety disorder [86]. Initially, vortioxetine was shown to be efficacious in reducing depressive and anxiety symptoms in patients with a major depressive disorder and high levels of anxiety (baseline total score of Hamilton Anxiety Rating Scale ≥ 20) [87], whereas two other meta-analyses showed that vortioxetine was not efficacious for anxiety [88,89]. However, following network meta-analysis of randomised controlled trials, vortioxetine was shown to be effective but was listed as the antidepressant with the lowest remission rates for generalised anxiety disorder [90]. Recent systematic review and meta-analysis suggested that there was uncertainty regarding the effectiveness of vortioxetine for anxiety symptom due to existing evidence arising from very low-quality studies [91].

In three placebo-controlled studies in adults with recurrent major depressive disorder, vortioxetine displayed clinically meaningful improvements in performance on two objective measures (the Digit symbol substitution test and the Rey auditory verbal learning test) that together cover a broad range of cognitive domains, including executive function, attention, processing speed, learning and memory [92,93,94]. In another meta-analysis, vortioxetine demonstrated the greatest improvements in a neurocognitive test that integrated several domains affected in major depressive disorder [95]. Notably, vortioxetine was the only antidepressant that provoked significant changes on task cognition among all antidepressant classes investigated [95]. A post hoc analysis demonstrated that vortioxetine showed general and multidomain benefits on cognitive performance, including executive function, attention/speed of processing and memory domains [96]. Therefore, the anxiolytic effects of vortioxetine did not reach expectations from preclinical findings, but vortioxetine is probably more useful in improving impairments of cognition or emotional perception than other monoamine transporter-inhibiting antidepressants.

In particular, enhancement of cognition with vortioxetine, in both subjective and objective functioning measures, was more pronounced in working patients, particularly in those whose job placed higher demands on executive functioning [94]. Indeed, patients prescribed vortioxetine recognised cognitive improvements that enhanced their workplace productivity [97]. Among the early onset of the antidepressive effect, anxiolytic effect and cognition-enhancing effect expected from the 5-HT7 inhibitory effect of vortioxetine (based on preclinical findings), the cognition-enhancing effect seems to be a characteristic effect of vortioxetine.

### 3.2. Lurasidone

A mood-stabilising antipsychotic agent, lurasidone possesses the highest binding affinity to 5-HT7 among antipsychotics [27] (Table 1). Several meta-analyses reported that lurasidone significantly improves positive and negative depressive symptoms [98,99,100,101]. Therefore, the general efficacy of lurasidone for the treatment of schizophrenia is considered to be comparable to that of other atypical antipsychotics [16]. Meta-analyses also demonstrated that lurasidone has antidepressant effects comparable to those of other mood-stabilising antipsychotics against major depressive disorder and bipolar depression [81,102]. The efficacy of lurasidone in the acute treatment of bipolar depression, as both monotherapy and adjunctive therapy to lithium/valproate, has been reported in clinical trials [103,104,105]. Like vortioxetine, the rapid onset of the antidepressive effects of lurasidone have not been demonstrated [78,79,80,81,82].

Several randomised control studies demonstrated that lurasidone improved comorbid anxiety symptom in schizophrenia, bipolar I and major depressive disorders. Lurasidone monotherapy (20~60 mg/day) improved depressive mood and concomitant anxiety in patients with bipolar I disorder [106]. Lurasidone monotherapy (20~80 mg/day) also improved depressive mood and anxiety in children and adolescents with bipolar depression [107]. Lurasidone (20–60 mg/day) was effective for mild and moderate-to-severe levels of anxieties for patients with major depressive disorder, suggesting the effectiveness of lurasidone on major depressive disorder with mixed features [80]. A clinical study suggested that lurasidone improved treatment-resistant schizophrenia (including clozapine-resistant schizophrenia) [108]. Notably, lurasidone improved speed of processing and executive function, which is a critical atypical antipsychotic-resistant cognitive domain [108]. In spite of limited evidence, the cognitive benefits of lurasidone in bipolar disorder and major depressive disorder was reported [109]. These demonstrations indicated the possibility that the effectiveness of lurasidone’s selective improvement of cognitive impairment is possibly modulated by specific transmission systems required for specific cognitive domains.

Considering the clinical findings regarding vortioxetine, both lurasidone and vortioxetine, at the minimum, might be classified in a distinct category of treatments that can be promised to improve cognitive function compared to conventional mood-stabilising antipsychotics and antidepressants. In contrast, the anxiolytic effects of lurasidone and vortioxetine displayed discrepancy, since vortioxetine has been evaluated to possess one of the lowest anxiolytic effects among monoamine transporter-inhibiting antidepressants [90], whereas lurasidone has demonstrated effectiveness treating anxiety symptoms in mood disorders and major depressive disorders with mixed features [80,106,107]. However, these evaluations of current meta-analyses probably could not measure the accurate efficacy of lurasidone in the treatment of schizophrenia, since dose–response meta-analyses demonstrated that 95% of the effective dose (ED95) of lurasidone for schizophrenia and depressive symptoms reached more than 160 mg/day, which was higher than the approved dose of lurasidone [101,110,111,112]. In other words, evaluation of the efficacy of lurasidone for schizophrenia needs to be supplemented with clinical data of higher doses than approval doses. The clinical findings of vortioxetine and lurasidone show that among the clinical effects expected in preclinical findings regarding 5-HT7 inhibition, only the improvement of cognitive impairment was demonstrated by both agents.

## 4. Intracellular Signalling Associated with 5-HT7

Four splice variants of 5-HT7 were identified. These were distinct in their carboxyl terminals due to introns in the 5-HT7 gene, including 5-HT7a, 5-HT7b and 5-HT7c in rodents, and 5-HT7a, 5-HT7b and 5-HT7d in humans [6,113,114,115,116,117]. All of these four splicing variants directly affect three intracellular signalling pathways via activations of Gαs, Gα12 and metalloproteinase-9 [4,46,118]. Significant differences among 5-HT7 splicing variants in localisation, ligand-binding affinity and adenylate cyclase activity have not been observed [6], whereas 5-HT7a isoform specifically activates the abovementioned cAMP-dependent signalling through the activation of type 1 and 8 adenylyl cyclase via Ca^2+^/calmodulin-dependent and Gs-independent signalling [119].

Activation of 5-HT7 enhances synthesis of cyclic adenosine monophosphate (cAMP) via activation of adenylyl cyclase [4]. Increasing cAMP activates signalling of both protein kinase A (PKA) and the exchange protein directly activated by cAMP (EPAC) [10,118,120]. These two signalling pathways affect various signalling transductions via phosphorylation of target proteins, leading to the propagation of the signalling to the next biochemical events. Subsequently, enhanced PKA stimulates cyclin-dependent kinase 5 (Cdk5) [10,118] and Ras [121,122], resulting in serine/threonine extracellular signal-regulated kinases (Erk) signalling activation [121,123]. Activation of EPAC also indirectly enhances Erk signalling [121,123]. The second signalling pathway regulates adenosine monophosphate-dependent protein kinase (AMPK) [42,47,48] via inhibitory PKA and stimulatory EPAC [124]. Furthermore, 5-HT7-induced Ras and EPAC signalling promote the activation of the mammalian target of rapamycin (mTOR) [125], but activated AMPK supresses mTOR signalling [126]. Activated signalling of Erk and mTOR by 5-HT7 enhances neurite outgrowth in embryonic brains [122,127].

**Figure 1:**
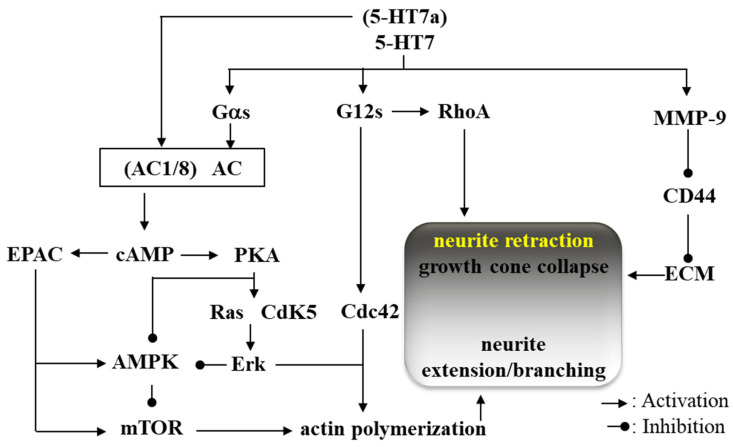
Intracellular signalling associated with 5-HT7. AC, adenylyl cyclase; AMPK, adenosine monophosphate-dependent protein kinase; cAMP, cyclic adenosine monophosphate; Cdc42, cell division cycle protein 42; Cdk5, cyclin-dependent kinase 5; EPAC, exchange protein directly activated by cAMP; Erk, extracellular-regulated kinase; mTOR, mammalian target of rapamycin; PKA, protein kinase A; RhoA, Ras homolog gene family member A; CD44, hyaluronic acid receptor; MMP-9, metalloproteinase 9; ECM, extracellular matrix.

Two other types of signalling associated with 5-HT7 were also identified: Gα12 [118] and metalloproteinase-9 (MMP9) [46]. It has been shown that 5-HT7/Gα12 activates cell division cycle protein 42 (Cdc42) [118,128] and activates signalling pathways associated with Gα12 [118]. In addition, it is recognized that 5-HT7/Gα12 activates both Ras homolog gene family member A (RhoA) and cell division cycle protein 42 (Cdc42) [118,128]. Another pathway associated with 5-HT7 activates MMP-9, which cleaves the extracellular domain of the hyaluronic acid receptor (CD44) resulting in subsequent detachment from the extracellular matrix (ECM) [46]. These physical interactions among extracellular matrices such as 5-HT7, CD44 and ECM, play fundamental roles in synaptic remodelling and dendritic spine elongation [46]. The detachment from ECM via CD44/MMP9 plays an initial role in both dendritic spine remodelling and synaptic pruning, followed by neurite retraction by RhoA and neurite extension/branching by mTOR, Erk and Cdc42 [122,127] (Figure 1).

It has been established that the serotonergic system plays crucial roles in the organisation of the neural system, such as generation of neurogenesis, cell migration, axon guidance, dendritogenesis, synaptogenesis and brain wiring during the development of the mature brain [129]. During the embryonic stage, 5-HT7 leads neurite outgrowth through activations of Cdk5 with mTOR [122,127]. Therefore, 5-HT7 contributes to the establishment and maintenance of neural connectivity and synaptic plasticity in early developmental stages [130]. In other words, the reorganization of dendritic morphology induced by 5-HT7 plays important roles in new synapse growth and initial neuronal network formation, which is the target of event-related structural and functional plasticity in the early developmental stage [131,132]. Chronic 5-HT7 activation promotes dendritic spine formation and increases the number of structurally intact synapses and the expression of α-amino-3-hydroxy-5-methyl-4-isoxazolepropionic acid (AMPA)/glutamate receptor [118]. Neural circuits can be remodelled, induced by reactions to physiological and pathological inputs well into adulthood and continuing to exhibit robust plasticity throughout the entire lifespan of individuals [133]. It is likely that 5-HT7 contributes to the modulation of synaptic plasticity and neuronal connectivity in the developing and mature brain. However, the 5-HT7 associated remodelling transforms their composition age-dependently. The impacts of 5-HT7/Gα12 signalling predominantly occur during the postnatal stages, but are reduced in older neurons, suggesting that neurite extension/branching induced by 5-HT7 is attenuated age-dependently [134]. Considered with the regulation system of neural remodelling associated with 5-HT7—e.g., neurite retraction (growth cone collapse) and neurite extension/branching—in the elderly brain, over-activation of 5-HT provides neurite retraction predominantly via 5-HT7/G12s and MMP-9 rather than through neurite extension/branching via 5-HT7/Gαs.

## 5. Effects of 5-HT7 on Neuronal Transmission

Behavioural study demonstrated that 5-HT7 knockout mice displayed impairments of contextual learning, seeking behaviour and allocentric spatial memory [135]. Electrophysiological study also demonstrated that 5-HT7 knockout mice exhibited impaired hippocampal long-term potentiation [64]. In addition, 5-HT7-induced activation of PKA signalling enhanced N-methyl-D-aspartate (NMDA)-evoked currents, resulting in the enhancement of population spike amplitude and bursting frequency in hippocampal CA1 and CA3 regions, respectively [136,137,138]. Furthermore, 5-HT7 activates hippocampal transmission postsynaptically due to enhanced phosphorylation of the AMPA/glutamate receptor induced by cAMP/cAMP response element-binding protein (CREB) signalling [139,140]. Additionally, 5-HT7 reversed long-term depression associated with metabotropic glutamate receptors (mGluR) [26]. These stimulatory effects of 5-HT7 on neuronal plasticity and excitability were observed in juvenile wild-type rodents, but not in elderly rodents [134]. These findings suggest that the molecular mechanisms underlying the positive action of 5-HT7 on cognition and memory are mediated by age-dependent regulation systems.

Activation of 5-HT7 during adolescence induced persistent upregulation of 5-HT7 [13]. Chronic exposure to methylphenidate during postnatal life and adolescence probably provides persistent structural rearrangements of the brain’s reward pathways associated with 5-HT7 [141]. During the pre- and postnatal periods, exposure to selective serotonin reuptake inhibitors generates long-term anxiety in adulthood without affecting the morphological alterations of the brain [142,143]. The molecular mechanism underlying 5-HT7-induced remodelling increases neurites and dendritic spine elongation via MMP-9/CD44 with Cdc42 in reversal learning and neuronal regeneration [46]. Therefore, the impacts of 5-HT7 on neuronal plasticity during early development are not limited to embryonic and early postnatal development but can also persist in adolescence and adulthood. Pathologically, activation of 5-HT7 during adolescence leads to increased dendritic arborisation in the nucleus accumbens, one of the most impactful neural circuits associated with the pathophysiology of schizophrenia [144,145,146]. Therefore, reorganization of dendritic morphology induced by 5-HT7 signalling provides new synapse growth and initial neuronal network formation in the hippocampus, which is the target of event-related structural and functional plasticity (memory) in the early developmental stage [131,132].

Although activation of hippocampal 5-HT7 contributes to the formation of learning and memory, suppression of 5-HT7, conversely, improved other cognitive components, such as executive function, which play important roles in quality of life. Traditionally, enhanced mesocortical dopaminergic transmission or dopamine release in the frontal cortex induced by blockade of D2 in the ventral tegmental area are considered to be key players in the improvement of positive and negative symptoms of schizophrenia [32]. Additionally, thalamocortical glutamatergic transmission are considered to play important roles in neurocognitive function [32,147,148,149]. 5-HT2A inhibition and/or 5-HT1A activation contributes to augmented dopamine release in the frontal cortex induced by D2 inhibition [150,151,152,153,154]. GABAergic disinhibition in the frontal cortex also contributes to enhanced dopamine release induced by some atypical antipsychotics, such as aripiprazole and clozapine [154,155]; however, lurasidone-induced dopamine release is not modulated by regional GABAergic disinhibition, similar to blonanserin, quetiapine, risperidone and zotepine [151,152,153,154]. These discrepancies in the frontal transmitter release profiles among 5-HT7 antagonistic antipsychotics (lurasidone, aripiprazole, clozapine, quetiapine, risperidone and zotepine) suggest that 5-HT7 antagonism probably does not play important roles in the enhanced dopamine release in the frontal cortex of these antipsychotics. Therefore, 5-HT7 antagonism is possibly not involved in effectiveness of the core (positive and negative) symptoms of schizophrenia.

Tonic hyperactivation of thalamocortical glutamatergic transmission was observed in schizophrenia, ADHD and autism [12,39,148,149,156,157,158,159,160,161,162,163,164,165]. Other 5-HT7 molecules, such as group II and III mGluRs and α2 adrenoceptor, which compensate thalamocortical glutamatergic transmission, have also been identified [149,156,157,159,164]. The behavioural importance of neuronal networks is affirmed by lesion studies demonstrating that lesions to hub regions are associated with task impairments across multiple functional domains [166,167,168]. Notably, the prediction of the thalamocortical pathway on task-specific cortical activity patterns was outperformed compared to the prediction of the cortical and hippocampal pathway [166,167,168]. The mediodorsal thalamic nucleus is reciprocally connected with the medial prefrontal cortex and receives glutamatergic inputs from the hippocampus [40,41,163,169,170]. The glutamatergic neurons in the mediodorsal thalamic nucleus were mainly suppressed but were activated by propagation of ripple burst during the non-rapid eye movement sleep phase [169,170,171]. Therefore, the mediodorsal thalamic nucleus plays important roles in memory processing during sleep and sensory integration during wakefulness [16,172,173,174,175]. Post-synaptic 5-HT7 on the glutamatergic neurons in the thalamus received serotonergic transmission from the dorsal raphe nucleus, resulting in attenuation of tonic activation of thalamocortical glutamatergic transmission [160,165]. Both lurasidone and vortioxetine suppress the 5-HT7-mediated hyperactivation of thalamocortical glutamatergic transmission via 5-HT7 blockade [12,39,40]. These findings indicate that inhibition of 5-HT7 in the mediodorsal thalamic nucleus contributes to sensory integration during wakefulness [16,173,174,175].

## 6. Therapeutic Potential in Other Disease and Disorders Based on the Preclinical Findings

### 6.1. Neurodevelopmental Disorders

Fragile X syndrome, which is caused by mutation of Fragile X mental retardation 1 (FMR1) gene, is a common neurodevelopmental disorder characterized by intellectual disability with autism [176]. Fragile X mental retardation protein (FMRP) is an mRNA-binding protein that plays important roles in the negative regulation of protein synthesis [176]. Loss of function of FMR1 leads to abnormal protein synthesis and overactivation of signalling via mGluR5 receptors, resulting in enhancement of long-term depression which, subsequently, induces aberrant synaptic plasticity [177,178]. FMR1 knockout mice exhibit sustained upregulation of mGluR-mediated long-term depression and decreasing density of the AMPA/glutamate receptor, whereas activation of 5-HT7 by selective 5-HT7 agonists, LP-211 and BA-10, reversed the pathological abnormalities to a normal level [26,123]. Therefore, it has been speculated that impaired cAMP-mediated signalling, which was observed in patients with Fragile X syndrome, is probably involved in the exaggerated generation of long-term depression [179].

Activation of 5-HT7 was a potential candidate target for relieving symptoms in patients with Rett syndrome. Rett syndrome is the second most common cause of mental retardation in females and plays a role in severe neurodevelopmental disorders such as breathing dysfunction, loss of coordination, abnormal eye and hand movements, epilepsy, aberrant sleeping behaviour and cognitive impairment [180]. The prime pathogenesis of Rett syndrome is known to be various genetic mutations in methyl CpG-binding protein 2 gene (MeCP2) on the X chromosome, cyclin-dependent kinase-like 5 (CDKL5), forkhead box G1 (FOXG1), WD repeat domain 45 (WDR45) or syntaxin binding protein 1 (STXBP1) [181,182]. Restoring MeCP2 function can normalise functional abnormalities of MeCP2 knockout mice, whereas overexpression of the MeCP2 gene led to neurological defects [183]. Therefore, recent preclinical studies explored the targets in MeCP2 downstream effectors and other signalling, including 5-HT7. Based on the reduced 5-HT7 expression in Rett syndrome models, the systemic administration of 5-HT7 agonist relieved the related symptoms, anxiety, environment-related exploratory behaviour and motor learning ability of Rett syndrome mice models [184,185]. Administration of 5-HT7 agonist also restored the inactivation of Rho GTPases’ downstream effectors, such as cofilin and the p21-activated kinase family, which regulate actin cytoskeleton polymerisation in Rett syndrome mice models [184,185]. Furthermore, subchronic administration of 5-HT7 agonist improved refined phenotypic alterations, locomotor response and synapse potentiation compared to non-treated model mice [185]. These studies, using genetic animal models of Rett syndrome, Mecp2-308 mice [184,185], demonstrated that subchronic administration of 5-HT7 agonist displayed persistent effects related to Rett syndrome, a result of increasing motivation regarding the development of 5-HT7 agonists for the treatment of Rett syndrome.

Association between 5-HT receptor gene polymorphism and autism spectrum disorder could not be detected by a transmission disequilibrium study [186], whereas it has been recognized that several antipsychotics used to alleviate aggressive behaviours in autism spectrum disorders, such as aripiprazole, lurasidone, pimozide and risperidone, are 5-HT7 inhibitors [16,187,188,189]. Considered alongside preclinical findings, these discrepancies suggest that any current knowledge regarding pathophysiology and pathophysiology of autism spectrum disorder is inadequate.

### 6.2. Neurodegenerative Diseases

Non-selectively activation of 5-HT receptors using 5-HT transporter inhibitors has shown little clinical benefit in neurodegenerative disorders [190,191]; however, several preclinical studies reported the attractive findings that 5-HT7 is a therapeutic candidate target for neurodegenerative disorders. Administration of 5-HT7 agonist also suppressed impairment of long-term potentiation and apoptosis in hippocampal streptozotocin-mediated neurodegeneration in murine models [192]. Amyloid-β induces neurotoxicity through several mechanisms including apoptosis, excitotoxicity and oxidative stress [193]; these were reverted by selective 5-HT7 agonist [194]. Amyloid-β is a key player in pathomechanisms of various neurogenerative diseases, such as Alzheimer’s disease, frontotemporal dementia, cerebral amyloid angiopathy, and cerebral amyloidosis, through multiple pathways [193,195]; however, the detailed roles of 5-HT7 in pathomechanisms of neurodegenerative disease associated with amyloid-β remain to be clarified [196,197].

### 6.3. Epilepsy

Various studies have reported on antiseizure activities—using audiogenic seizures in DBA/2J mice, or the absence of seizures in WAG/Rij rats—following the administration of selective and non-selective antagonists [198,199]. These studies demonstrated that SB269970 and AS-19 decreased and increased the frequency of pilocarpine-induced temporal lobe epilepsy seizures in rat models, respectively [200]. The same report also demonstrated that expression of 5-HT7 in brain tissue extracted from patients with temporal lobe epilepsy and its expression in pilocarpine-induced temporal lobe epilepsy rat models increased in comparison to normal subjects [200]. These findings suggested the possibility that pharmacologically, 5-HT7 inhibitors possess a therapeutic potential for epilepsy. Contrary to pharmacological findings, 5-HT7 knockout mice were more sensitive to pentylenetetrazole, cocaine and NMDA-induced seizures compared to wild-types [201].

### 6.4. Cartinoma

There are some findings indicating that 5-HT7 modulation may be therapeutic targets for the treatment of cancer. Notably, it has been anticipated that 5-HT7 antagonists would be useful for the treatment of hepatocellular cancer and small-intestinal neuroendocrine neoplasms, since 5-HT7 contributes to hepatocyte proliferation [18,19,20]. Serotonergic function activates proliferation of hepatocellular cancer and the expression of β-catenin, which plays an important role in the pathomechanisms of hepatocellular cancer via the activation of 5-HT7. The expression of 5-HT7 increased in hepatocellular cancer cell lines, but the 5-HT7 antagonist SB258719 suppressed 5-HT-induced increase in β-catenin levels and cell viability. Furthermore, SB258719 also suppressed proliferation and tumour growth. When small-intestinal neuroendocrine neoplasms, which are tumours derived from enterochromaffin cells, metastasize to the liver, the liver produces 5-HT, resulting in hyperactivation of 5-HT7 in the liver [20]. Hyperactivation of 5-HT7 in the liver leads to secretion of the growth factor IGF-1, which accelerates proliferation of metastatic tumour cells. Based on these findings, 5-HT7 inhibition is considered to be a candidate target for the treatment of hepatic metastasis cancers. It has been reported that 5-HT7 expression increased in certain types of breast cancers; this increase was amplified with tumour grade [202]. Indeed, SB269970 suppressed tumour formation in an MDA-MB-231 cancer cell [21].

## 7. Impacts of 5-HT7 Inhibition on Metabolic Complication in Patients with Intake of Atypical Antipsychotics

The mortality gap between patients with schizophrenia and the general population has been growing [203]. Indeed, the life expectancy of patients with schizophrenia is 16 years shorter than the general population, with more than 30% of excess deaths attributable to metabolic complications [203]. The prevalence of obesity in patients with psychiatric disorders (up to 60%) has been reported to be twice that in the general population [204]. It has been established that the hyperactivation of AMPK in the hypothalamus, induced by H1 and 5-HT2A blockade, is involved in the pathophysiology of antipsychotic-induced weight gain and metabolic complication [205]. Conversely, activation of AMPK activity in the peripheral organs is one of the most established targets for treating insulin-resistant diabetes [206,207], whereas hypothalamic AMPK seems to play fundamental roles in regulating both sides of the energy balance equation (feeding and energy expenditure) in the entire body [206]. Blockade of several monoamine receptors, such as H1 and 5-HT2A, decreases inositol trisphosphate (IP3) synthesis, which enhances calcium-induced calcium release (CICR) via the activation of the IP3 receptor [208,209,210], leading to suppressed adenosine triphosphate (ATP) synthesis [208,211]. The relatively increasing AMP or decreasing ATP intracellular levels activate AMPK activity [211]. Indeed, antipsychotics with high-risk for weight gain, such as clozapine, olanzapine, quetiapine and zotepine (possessing high binding affinity to H1 and 5-HT2A), enhance hypothalamic AMPK signalling, whereas lower-risk antipsychotics, such as lurasidone and brexpiprazole (possessing high binding affinity to 5-HT2A but lower affinity to H1) suppress AMPK signalling [42,47,48,205,212,213]. Additionally, both lurasidone and brexpiprazole possess high binding affinity with 5-HT7 inhibitors and suppress cAMP synthesis and AMPK signalling [42,48].

The detailed mechanisms of the suppression of AMPK signalling induced by 5-HT7 inhibition remained to be clarified, but decreased EPAC signalling is predicted to be one of the key players. Considering the binding profile of clozapine, which also posssesses relatively high affinity to 5-HT7, 5-HT7 inhibition cannot fully suppress the weight gain induced by H1 inhibition, but, at the minimum, it may suppress the activation of AMPK activity induced by 5-HT2A inhibition.

## 8. Conclusions

The present review introduced the neuropharmacological findings of 5-HT7 modulation demonstrated by preclinical and clinical studies. The expected clinical effects of 5-HT7 inhibition on schizophrenia, mood and anxiety disorders by preclinical studies probably have not been able to contribute to the improvement of the core symptoms of these disorders; however, 5-HT7 inhibition possibly improves cognitive impairments in patients with schizophrenia and mood disorders compared to predictions from preclinical studies. Furthermore, it is suggested that 5-HT7 inhibition seems to have a mitigating effect on antipsychotic-induced weight gain and metabolic complication. Preclinical studies for the clinical application of 5-HT7 agonists for neurodevelopmental disorders and neurodegenerative diseases are currently underway. Until recently, several findings of these preclinical studies suggest that 5-HT7 modifications can serve two independent targets. The first is that 5-HT7 inhibition ameliorates the dysfunction of inter-neuronal transmission in mature networks. The other is that activation of 5-HT7 can improve transmission dysfunction due to microstructure abnormality in the neurotransmission network, which could not be affected by conventional therapeutic agents, via modulating intracellular signalling during the neurodevelopmental stage or through the loss of neural networks with aging. Therefore, the 5-HT7-modulating medications may be able to acquire a more practical treatment option via selectively using enhancement and suppression in an age- or pathological condition-dependent manner, rather than a binary choice.

## Data Availability

Not applicable.
